# 

**Published:** 1894-02

**Authors:** 


### CONTENTS.

### *EDITORIAL.*

PAGE

The Responsibility of Selecting the Ideal Simillimum, . . . . 33

### *MISCELLANEOUS.*

Commentaries on *The Organon.* B. Fincke, M. D., . . . . . 35

A Clinical Experience. Adolph Lippe, M. D., . . . . . . 40

Scrofula. Proceedings of I. H. A., . . . . . . . . 43

Discussions upon Surgery. Proceedings of I. H. A., . . . . 47

Gleanings. F. H. Lutze, M. D., . . . . . . . . . . 52

Provings and Clinical Observations with High Potencies. Malcolm Macfarlan, M. D., . . . . . . . . . . 56

Relation of Pain to Swallowing. A. McNeil, M. D., . . . . . . 62

Significant Notes, . . . . . . . . . . . 62

Complication or Coincidence? . . . . . . . . 62

Bee-Virus for Acute Rheumatism, . . . . . . . 63

### *NOTES AND NOTICES.*

Stearns' Calendar for 1894, . . . . . . . . . . 64

Remedies having Cold Saliva. W. A. Yingling, M. D., . . . . 64

